# Altered oral and gut microbiota and its association with SARS-CoV-2 viral load in COVID-19 patients during hospitalization

**DOI:** 10.1038/s41522-021-00232-5

**Published:** 2021-07-22

**Authors:** Yongjian Wu, Xiaomin Cheng, Guanmin Jiang, Huishu Tang, Siqi Ming, Lantian Tang, Jiahai Lu, Cheng Guo, Hong Shan, Xi Huang

**Affiliations:** 1 Center for Infection and Immunity, The Fifth Affiliated Hospital, Sun Yat-sen University, Zhuhai, Guangdong China; 2 School of Public Health, Sun Yat-sen University, Guangzhou, Guangdong China; 3 Guangdong Provincial Engineering Research Center of Molecular Imaging, Guangdong Provincial Key Laboratory of Biomedical Imaging, and Department of Interventional Medicine, The Fifth Affiliated Hospital, Sun Yat-sen University, Zhuhai, Guangdong China; 4 Southern Marine Science and Engineering Guangdong Laboratory, Zhuhai, Guangdong China; 5 National Clinical Research Center for Infectious Disease, Shenzhen Third People’ s Hospital; The Second Affiliated Hospital of Southern University of Science and Technology, Shenzhen, Guangdong China; 6 Department of Clinical Laboratory, The Fifth Affiliated Hospital of Sun Yat-sen University, Zhuhai, Guangdong China; 7 Center for Infection and Immunity, Mailman School of Public Health, Columbia University, New York, NY USA

**Keywords:** Clinical microbiology, Microbiome

## Abstract

The human oral and gut commensal microbes play vital roles in the development and maintenance of immune homeostasis, while its association with susceptibility and severity of SARS-CoV-2 infection is barely understood. In this study, we investigated the dynamics of the oral and intestinal flora before and after the clearance of SARS-CoV-2 in 53 COVID-19 patients, and then examined their microbiome alterations in comparison to 76 healthy individuals. A total of 140 throat swab samples and 81 fecal samples from these COVID-19 patients during hospitalization, and 44 throat swab samples and 32 fecal samples from sex and age-matched healthy individuals were collected and then subjected to 16S rRNA sequencing and viral load inspection. We found that SARS-CoV-2 infection was associated with alterations of the microbiome community in patients as indicated by both alpha and beta diversity indexes. Several bacterial taxa were identified related to SARS-CoV-2 infection, wherein elevated *Granulicatella* and *Rothia mucilaginosa* were found in both oral and gut microbiome. The SARS-CoV-2 viral load in those samples was also calculated to identify potential dynamics between COVID-19 and the microbiome. These findings provide a meaningful baseline for microbes in the digestive tract of COVID-19 patients and will shed light on new dimensions for disease pathophysiology, potential microbial biomarkers, and treatment strategies for COVID-19.

## Introduction

The ongoing COVID-19 pandemic caused by the severe acute respiratory syndrome coronavirus 2 (SARS-CoV-2) has jeopardized global public health and safety. As of June 21, 2021, the COVID-19 pandemic has infected more than 179 million people, resulting in over 3.8 million causalities globally. Transmission of SARS-CoV-2 has commonly occurred via breathing or direct contact with virus-containing droplets and aerosols from infected people through coughing and sneezing. When the SARS-CoV-2 particle reaches the nasal cavity of the host, it enters the host epithelial cells through the angiotensin-converting enzyme 2 (ACE2) receptor, which is prominently presented on epithelial cells lining in the respiratory and digestive tract systems^1,2^. Most infections lead to a prompt innate immune response and the virus gets eradicated or controlled quickly, manifesting none or mild symptoms. Whereas in some patients, viruses located in the upper airway replicate further toward the lower respiratory tract to activate enhanced pro-inflammatory responses, therefore resulting in severe outcomes including acute respiratory syndrome, organ malfunctioning, and even death^3^. Not limited to the respiratory tract, SARS-CoV-2 is known to target different organ systems^4^. For instance, around 55% of patients were observed with prolonged viral RNA present in the feces, even weeks after the viral clearance in their respiratory tract^5^. It has been reported that abnormal immune response, comorbid conditions, and advanced age are risk factors linked to COVID-19 severity. However, these factors are insufficient to offer a satisfactory explanation of all patients’ severe disease outcomes. As global mass vaccination and proven effective therapy for COVID-19 remain unclear, explorative efforts towards new perspectives of protection and therapeutic approaches for COVID-19 are indispensable^6,7^.

The human microbiome is important for developing and maintaining immune homeostasis and it is known that microbiota imbalance or dysbiosis are highly associated with various diseases. The intestinal tract and oral cavity, with the largest and second-largest microbiota in the human body, play significant roles in the pathogenesis of infectious disease. Previous studies have reported that oral-lung microbes can influence the outcome of many infectious diseases by regulating the host mucosal immunity^8–10^. Intestinal flora can affect the occurrence and progression of viral infection through the gut–lung axis^11–13^. Conversely, the indigenous microbiome could be disturbed by a viral infection, leading to alterations in susceptibility and disease severity through dysbiotic community structure and function^14^. The unequivocal association between influenza viral and bacterial co-infection and disease severity has been proved in early studies^15^. Likewise, evidence has suggested that SARS-CoV-2 infection could predispose patients to bacterial co-infections and superinfections, resulting in increased disease severity and mortality^14^. Further, the dysbiosis of influenza-infected individuals progresses toward microbiota homeostasis, coinciding with viral clearance and recovery, suggesting that the health status of microbial flora is likely a fine indication of disease recovery^16^. Zuo and colleagues have also suggested that fecal microbiomes from COVID-19 patients were characterized by proliferation of opportunistic pathogens and depletion of favorable commensals compared to healthy controls with the shot-gun metagenomics approach^17^; however, this study was limited by its size of subjects used. Moreover, validation of the profiles at the genus level is currently suggested to be performed through 16S rRNA gene microbial profiling to evaluate the presence of undetected microbes in the marker gene-based profiles^18^. Accumulated evidence for the oral–gut axis has revealed its role in modulating the pathogenesis process in numerous diseases^19,20^. It is compelling to look into the oral and intestinal microbiome combined with SARS-CoV-2 infection and the crosstalk among them, which may provide an improved understanding of the initiation of viral infection and the path of disease deterioration.

## Results

### Overview of microbial composition in the study subjects

**Fig. 1:**
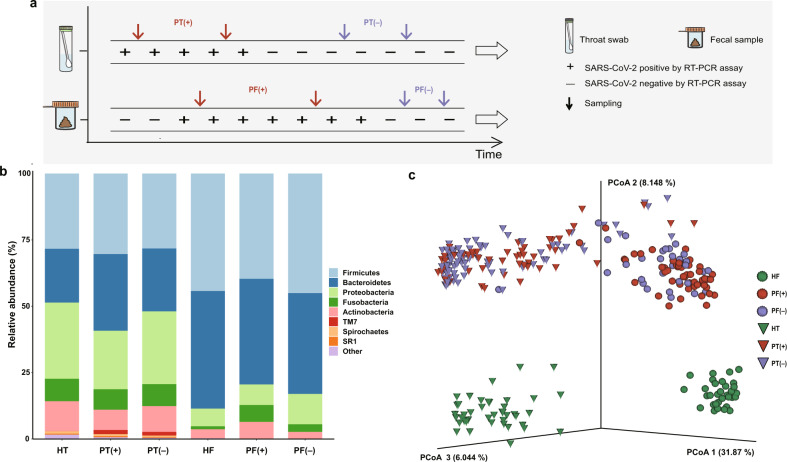
The study design and variations of the oral and gut microbiota in COVID-19 patients. **a** Graphic representation of study design and sample collection. PT(+), throat swab samples from COVID-19 patients during the positive viral RNA test period; PT(−), throat swab samples from COVID-19 patients during the negative viral RNA test period; PF(+), fecal specimens from COVID-19 patients during the positive viral RNA test period; PF(−), fecal specimens from COVID-19 patients during the negative viral RNA test period. **b** Bacterial composition at the phylum level among groups. Sample size (*n*) for each group: HT, *n* = 44; PT(+), *n* = 52; PT(−), *n* = 88; HF, *n* = 32; PF(+), *n* = 50; PF(−), *n* = 31. HT, throat swab samples from the healthy controls; HF, fecal specimens from the healthy controls. **c** PCoA plot based on the unweighted UniFrac distance depicting differences in the bacterial community among groups.

A total of 53 patients diagnosed with COVID-19 and 76 healthy individuals were included in this study. As indicated in Fig. 1a, serial throat swab samples and fecal specimens were collected from COVID-19 patients during hospitalization, covering both the positive viral RNA test period (P-VRTP) and the negative viral RNA test period (N-VRTP). Depending on the sample availability, a total of 140 throat swab samples, including 52 during the P-VRTP [PT(+)] and 88 during the N-VRTP [PT(−)], and a total of 81 fecal samples, including 50 during the P-VRTP [PF(+)] and 31 during the N-VRTP [PF(−)] were collected from hospitalized patients (P: patient). In addition, 44 throat swab samples (HT) and 32 fecal samples (HF) from sex and age-matched healthy individuals (H: healthy individual) were included in our study as controls. The sample distribution, demographics, and relevant clinical information about the subjects recruited in the study were summarized in Supplementary Table 1.

To characterize the microbiotas in those aforementioned samples, we sequenced the V3–V4 region of the bacterial 16S rRNA gene. As expected, our data confirmed a distinct microbiota composition between oral and gut samples^21^. The three most abundant phyla *Firmicutes*, *Proteobacteria*, and *Bacteroidetes* accounted for 79.3% of the community in the oral samples. The two dominant phyla *Firmicutes* and *Bacteroidetes* accounted for 83.7% of the bacterial community in the gut samples (Fig. 1b). The difference was further illustrated by the principal coordinates analysis (PCoA) plot and Permutational multivariate analysis of variance (PERMANOVA) with unweighted UniFrac distance calculated at the sequence feature level. The microbiota composition of the oral and gut samples diverged from each other along the first axis in which 31.87% of the total variance was explained, indicating that the largest source of variation was the sample types (Fig. 1c). Similarly, the PERMANOVA result indicated 30.80% of the total variance was explained by the sample types (oral samples vs. gut samples, PERMANOVA, *R*^2^ = 0.308, *p* < 0.001). More importantly, samples from patients and healthy individuals seemed to possess strong dissimilarities for both oral (PERMANOVA, *R*^2^ = 0.175, *p* < 0.001) and gut microbiome communities (PERMANOVA, *R*^2^ = 0.196, *p* < 0.001), suggesting overwhelming shifts of microbiome structure were gained after SARS-CoV-2 infection in both oral cavity and feces. Meanwhile, the microbial compositions of COVID-19 patients collected during the P-VRTP and the N-VRTP presented no significant discrepancy in oral samples (PERMANOVA, *R*^2^ = 0.012, *p* = 0.080) or in gut samples (PERMANOVA, *R*^2^ = 0.016, *p* = 0.139). Due to their distinctive characteristics, the oral and gut microbiome profiles and their potential roles in SARS-CoV-2 infection were addressed in separate sections followed.

### Alterations of the oral microbiota in patients

**Fig. 2:**
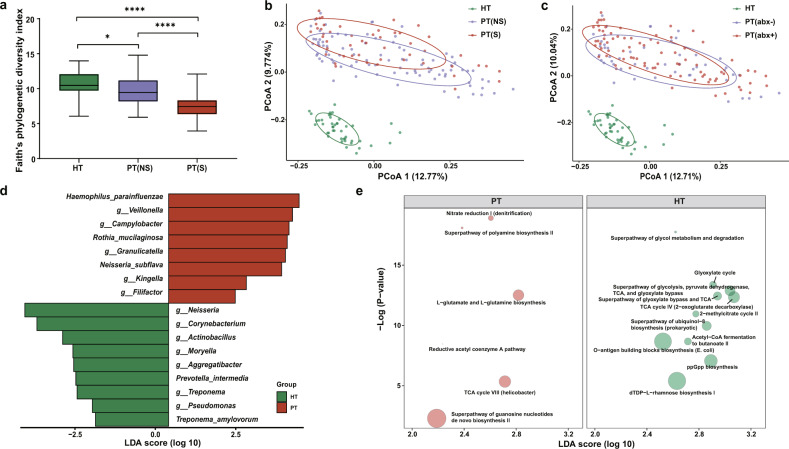
Altered oral microbiota in COVID-19 patients. **a** Species diversity differences among the HT (*n* = 44), PT(NS) (*n* = 90), and PT(S) (*n* = 50) groups were estimated by Faith’s phylogenetic diversity index. *p < 0.05, ****p < 0.0001. *p*-values were obtained using one-way ANOVA followed by Tukey’s multiple comparisons test. The line in the middle of the box, bound of the box and whiskers represent the median, 25th–75th percentiles, and min-to-max values, respectively. PT(NS), throat swab samples from the non-severe patients group; PT(S), throat swab samples from the severe patients group. **b** PCoA plot based on unweighted UniFrac distances showing microbial structural clustering among the HT, PT(NS), and PT(S) groups. Ellipses represent 68% confidence intervals. **c** PCoA plot according to unweighted UniFrac distances displaying bacterial structural discrimination among the HT (*n* = 44), PT(abx−) (*n* = 49), and PT(abx+) (*n* = 91) groups. Ellipses represent 68% confidence intervals. PT(abx−), throat swab samples from patients without antibiotic interference; PT(abx+), throat swab samples from patients with antibiotic interference. **d** Bacterial taxa identified a significant difference between HT (*n* = 44) and PT (*n* = 140) groups by LEfSe. Seventeen taxa were validated using MaAsLin2 adjusting for covariates. PT throat swab samples from patients, LDA linear discriminant analysis. **e** Differentially abundant MetaCyc pathways identified with the functional analysis result of metagenomes. Only the pathways identified by both LEfSe and MaAsLin2 were presented. Circle size represents the relative abundance of pathways.

Given the observation that the SARS-CoV-2 infection may impact the structure of the microbiome (Fig. 1c), we first investigated the effects of different disease severity conditions and treatment therapies on the oral samples from patients. The diversity metrics among oral microbiomes from the healthy controls (HT), patients with the non-severe condition [PT(NS)], and patients with the severe condition [PT(S)] during hospitalization were compared. Alpha diversity of Faith’s phylogenetic index revealed that the oral microbial diversity was decreased in COVID-19 patients compared to the healthy controls (ANOVA, PT(NS) vs. HT, *p* = 0.016; PT(S) vs. HT, *p* < 0.0001). Further, a significant deduction of diversity was observed in the PT(S) group when comparing to the PT(NS) group (ANOVA, *p* < 0.0001, Fig. 2a). Similarly, inter-individual beta diversity unweighted UniFrac dissimilarity revealed that the microbiomes of the PT(NS) group (PERMANOVA, *R*^2^ = 0.238, *p* < 0.001) and the PT(S) group (PERMANOVA, *R*^2^ = 0.233, *p* < 0.001) clustered apart from that of the healthy controls. We also found a significant dissimilarity between the PT(S) group and the PT(NS) group (PERMANOVA, *R*^2^ = 0.049, *p* < 0.001, Fig. 2b). The significant differences were partially attributed to the different dispersions between the PT(S) group and the PT(NS) group (PERMDISP, *p* = 0.002). In contrast, the difference in dispersion between the HT group and the PT(NS) group was not significant (PERMDISP, *p* = 0.166). We also investigated the impact of clinical antibiotic usage. The PCoA based on unweighted UniFrac dissimilarity demonstrated that bacterial communities in the HT group, the patients without antibiotic interference [PT(abx−)] group and the patients with antibiotic interference [PT(abx+)] group were mutually separated (PERMANOVA, PT(abx−) vs. HT, *R*^2^ = 0.258, *p* = 0.001; PT(abx+) vs. HT, *R*^2^ = 0.204, *p* = 0.001; PT(abx−) vs. PT(abx+), *R*^2^ = 0.016, *p* = 0.016; Fig. 2c). To identify potential microbial biomarkers associated with COVID-19 patients, we employed a combined approach with LEfSe and MaAsLin2 to minimize the influence of confounding factors. Seventeen significantly different taxa were identified in the oral microbiome between the healthy controls and COVID-19 patients (HT and PT, Fig. 2d and Supplementary Table 2). Specifically, significant decreases of *Neisseria*, *Corynebacterium*, *Actinobacillus*, *Moryella*, *Aggregatibacter*, *Treponema,* and *Pseudomonas* at the genus level, as well as *P. intermedia* and *T. amylovorum* were observed in the patients comparing to the controls. In contrast, *Veillonella*, *Campylobacter*, *Granulicatella*, *Kingella,* and *Filifactor* at the genus level, as well as *H. parainfluenzae*, *R. mucilaginosa,* and *N. subflava* were enriched in the oral microbial communities of COVID-19 patients compared to those of the controls. A similar comparison was conducted between the PT(NS) and the PT(S) groups to assess the impact of disease severity on bacteria (Supplementary Table 3). Bacterial taxa including *Treponema*, *Aggregatibacter,* and *P. intermedia* were identified further depleted in the PT(S) group, implying their associations with disease severity. The comparison between the controls and the PT(abx–) group was carried out to assess the impact of infection on bacteria by excluding the antibiotics interference (Supplementary Table 3). Lastly, the potential functional consequence resulted from the drastic taxa compositional alteration was evaluated. The global pattern from Procrustes analysis displayed a good-fit correlation between the oral microbial community composition and microbial function (Mantel, rho = 0.403, *p* = 0.001, Supplementary Fig. 1a). The significant difference in functional pathways of the oral microbial communities between COVID-19 patients and the controls was shown in Fig. 2e. Notably, the top four depleted pathways in patients were involved in the TCA cycle used by all aerobic organisms to generate energy, indicating a disturbed microbial community.

### Alterations of the gut microbiota in patients

**Fig. 3:**
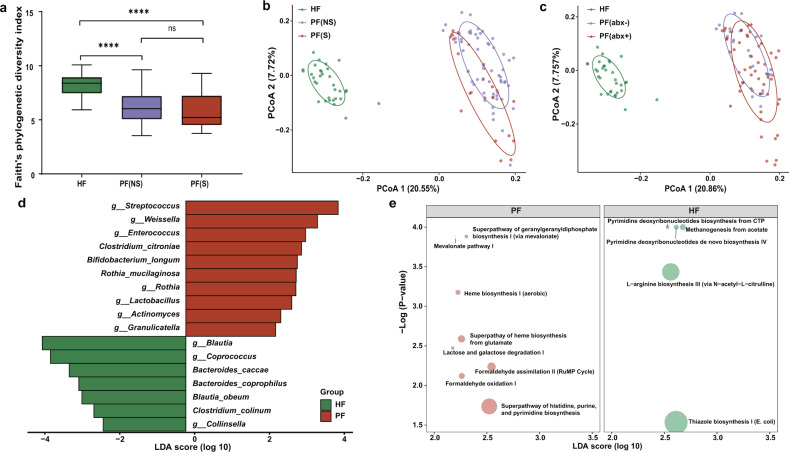
Altered gut microbiota in COVID-19 patients. **a** Species diversity differences among the HF (*n* = 32), PF(NS) (*n* = 64), and PF(S) (*n* = 17) groups were estimated by Faith’s phylogenetic diversity index. ns: not significant, *p < 0.05, ****p < 0.0001. *p*-values were obtained using one-way ANOVA followed by Tukey’s multiple comparisons test. The line in the middle of the box, bound of the box and whiskers represent the median, 25th–75th percentiles, and min-to-max values, respectively. PF(NS), fecal specimens from the non-severe patient group; PF(S), fecal specimens from the severe patient group. **b** PCoA plot based on unweighted UniFrac distances showing microbial structural clustering among the HF, PF(NS), and PF(S) groups. Ellipses represent 68% confidence intervals. **c** PCoA plot according to unweighted UniFrac distances displaying bacterial structural discrimination among the HF (*n* = 32), PF(abx−) (*n* = 30), and PF(abx+) (*n* = 51) groups. Ellipses represent 68% confidence intervals. PF(abx−), fecal specimens from patients without antibiotic interference; PF(abx+), fecal specimens from patients with antibiotic interference. **d** Bacterial taxa identified a significant difference between the HF (*n* = 32) and PF (*n* = 81) groups by LEfSe. Seventeen taxa were validated using MaAsLin adjusting for covariates. PF fecal samples from patients, LDA linear discriminant analysis. **e** Differentially abundant MetaCyc pathways identified with the functional analysis result of metagenomes. Only the pathways identified by both LEfSe and MaAsLin2 were presented. Circle size represents the relative abundance of pathways.

Next, we explored whether dysbiosis occurred in the gut microbiome. Faith’s phylogenetic diversity index showed that its diversity was significantly lower in patients with the non-severe condition [PF(NS)] (ANOVA, *p* < 0.0001) and patients with the severe condition [PF(S)] (ANOVA, *p* < 0.0001) in comparisons to the healthy controls (HF, Fig. 3a). The diversity of the PF(NS) group was slightly higher than the PF(S) group (ANOVA, *p* = 0.73). Besides, the PCoA plot with unweighted UniFrac distances revealed a cluster separation in the fecal microbiota between PF(NS) and PF(S) (PERMANOVA, *R*^2^ = 0.067, *p* < 0.001), and between the patients and the healthy controls (PERMANOVA, PF(NS) vs. HF, *R*^2^ = 0.232, *p* < 0.001; PF(S) vs. HF, *R*^2^ = 0.267, *p* < 0.001, Fig. 3b). Unlike the oral microbiome, the gut microbiota in the PF(NS) group seemed more impacted after infection, as reflected by its greater statistical dispersion than the HF group (PERMDISP, *p* = 0.002). Moreover, the gut microbiota among patients with antibiotic interference [PF(abx+)], patients without antibiotic interference [PF(abx−)], and the HF group exhibited mutual clustering separation with unweighted UniFrac distances based PCoA plot (PERMANOVA, PF(abx−) vs. HF, *R*^2^ = 0.262, *p* = 0.001; PF(abx+) vs. HF, *R*^2^ = 0.230, *p* = 0.001; PF(abx−) vs. PF(abx+), *R*^2^ = 0.030, *p* = 0.002; Fig. 3c). In terms of potential microbial biomarkers, we identified 17 bacterial taxa again with confounding covariates adjusted. Concretely, decreased taxa included *Blautia*, *Coprococcus*, and *Collinsella* at the genus level, and *B. caccae*, *B. coprophilus*, *B. obeum,* and *C. colinum* species; and increased taxa included *Streptococcus*, *Weissella*, *Enterococcus*, *Rothia*, *Lactobacillus*, *Actinomyces*, and *Granulicatella* at the genus level as well as *C. citroniae*, *B. longum,* and *R. mucilaginosa* species (Fig. 3d and Supplementary Table 4). More interestingly, two normal components of the respiratory tract flora, *Granulicatella* and *R. mucilaginosa*, were significantly increased in the patients for both oral and gut samples, and *R. mucilaginosa* seemed further associated with the disease severity in the fecal samples (Supplementary Fig. 2). Additional analysis focusing on the bacteria changes associated with disease severity and antibiotics usage in fecal samples were described in Supplementary Table 5. Notably, no difference in the bacterial taxa was identified between the PF(NS) group and the PF(S) group. Similar to the oral microbiome, the global pattern from Procrustes analysis displayed a good-fit correlation between the microbial composition and predicted function profile in the gut microbiota community (Mantel, rho = 0.268, *p* = 0.001, Supplementary Fig. 1b). Also, the major distinct functional pathways associated with SARS-CoV-2 infection were shown in Fig. 3e, including two depleted pyrimidine deoxyribonucleotides biosynthesis pathways involved in viral suppression through innate immunity in patients^22^.

### Relationship between SARS-CoV-2 virus and bacterial species

**Fig. 4:**
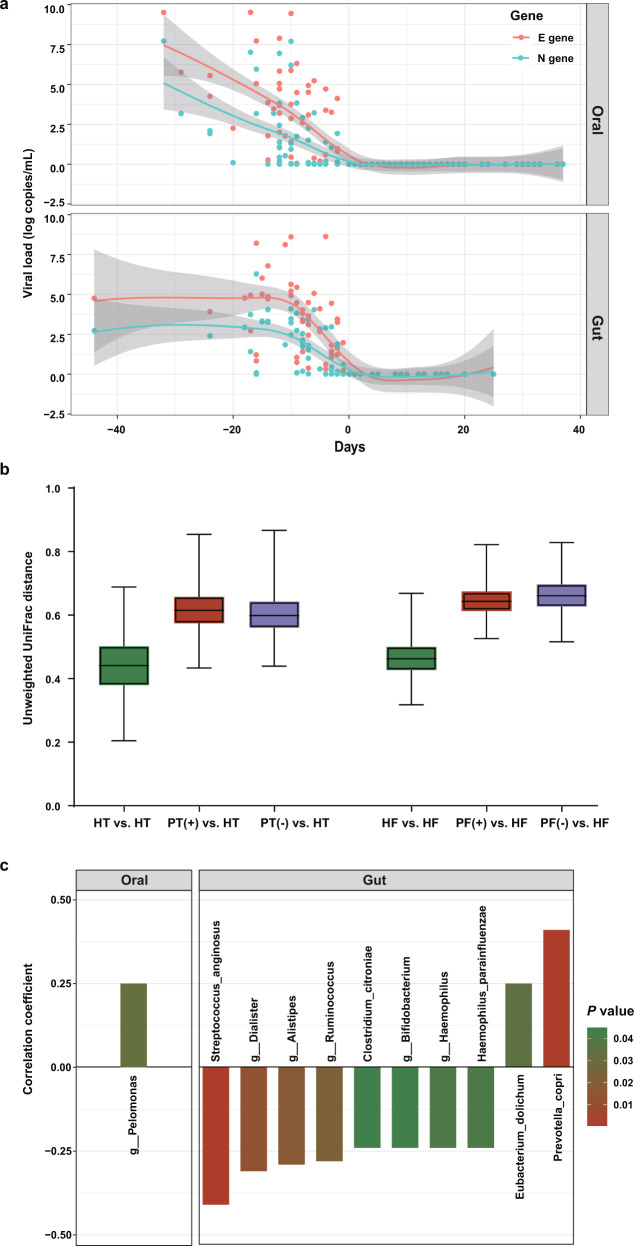
Associations between oral/gut microbiota disturbance and SARS-CoV-2 viral loads in hospitalized patients. **a** Longitudinal changes of viral loads in throat swab samples and fecal specimens from COVID-19 patients. Day 0 indicated the negative conversion time of SARS-CoV-2 RNA. The red line and blue line indicated longitudinal changes in viral loads represented by the abundances of the E gene and N gene, respectively. **b** Boxplots showing unweighted UniFrac distances within and between groups. **c** The longitudinal viral load represented by the abundances of the E gene significantly correlated with the timepoint-matched relative abundances of different taxa in throat swab and fecal samples separately. *p*-values were obtained using CCLasso.

The viral loads in the throat swab and fecal samples spanning the full collection period were compared. The median time span of the throat swabs collection was 10 (IQR: 6–12) days for P-VRTP and 10 (IQR: 3–18) days for N-VRTP. The median time span of the fecal samples collection was 8 (IQR: 3.75–14) days for P-VRTP and 8 (IQR: 2–12) days for N-VRTP. The viral copy numbers derived from the E gene showed a relatively higher abundance over the numbers derived from the N gene (Fig. 4a). As E-gene and N-gene copy numbers showed a strong correlation therefore both can accurately reflect the abundance of viral copy (throat swab samples: Spearman rho = 0.872, *p* < 0.001; fecal samples: Spearman rho = 0.923, *p* < 0.001). The overall viral loads in throat swab samples seemed equally abundant with fecal specimens collected from patients during the P-VRTP, which is consistent with the previous studies^23^. Based on unweighted UniFrac distance, it was found that the differences in beta diversity between the HT and PT(+) groups (PERMANOVA, *R*^2^ = 0.234, *p* = 0.001), the HT and PT(−) groups (PERMANOVA, *R*^2^ = 0.218, *p* = 0.001), were significant from each other. Likewise, unweighted UniFrac dissimilarity revealed that the microbiomes of the PF(+) group (PERMANOVA, *R*^2^ = 0.233, *p* = 0.001) and the PF(−) group (PERMANOVA, *R*^2^ = 0.250, *p* = 0.001) were significantly different from that of the healthy controls. During the N-VRTP, we observed a microbial community recovery towards the healthy controls, that is, unweighted UniFrac distance between PT(−) vs. HT was lower than that between PT(+) vs. HT (Fig. 4b). Compared to the oral microbiome, this recovery trend in the microbiome in the gut was less evident, which may be attributed to a relatively shorter time span of sample collection and microbiome robustness in the fecal samples. Additionally, we investigated whether the SARS-CoV-2 viral load was associated with any oral and gut bacterial species. In the oral microbiota, *Pelomonas* was identified as positively correlated with the viral load of SARS-CoV-2 (Fig. 4c). While in the gut microbiota, *P. copri* and *E. dolichum* were identified positively correlated with the viral load of SARS-CoV-2, and *S. anginosus*, *Dialister*, *Alistipes*, *Ruminococcus*, *C. citroniae*, *Bifidobacterium*, *Haemophilus*, and *H. parainfluenzae* were identified negatively correlated with the viral load of SARS-CoV-2.

## Discussion

The oral and gut microbiome offers a range of valuable properties to the host. Several most significant contributions of these microbes are to boost metabolism, improve digestive health and strengthen resistance against pathogens. Furthermore, the complex crosstalk between commensal microbes and different body systems is essential for the functioning of the immune system^24^.

Our data revealed profound alterations in both oral and gut microbiomes, which were reflected in the dramatic changes in community structure, potential bacterial marker species, and predicted functional profile. The decline in commensal bacterial diversity has been considered as a key dysbacteriosis indicator in several diseases^25^. Coherently, the oral and gut microbiome of COVID-19 patients in our study exhibited decreased diversities compared to the healthy controls. This trend of microbial imbalance in patients with severe conditions compared to non-severe patients was also observed in both sample types, though the alpha diversity was not significant (ANOVA, *p* = 0.73) in the fecal samples. Moreover, the microbiomes from the severe and the non-severe patients were partitioned into two clusters in both microbial populations. Overall, diversity results of impaired microbiota suggested the strong association between the microbiome community complexity and the disease severity in COVID-19 patients. Though experimental validation is needed, our result has highlighted the possibility of personalized microbiota to affect the disease outcome of COVID-19 patients^26^.

Numerous studies have reported high occurrences of bacterial co-infection in hospitalized COVID-19 patients, and the odds of the bacterial infection get even higher for ICU patients^27,28^. Among the list of eight bacterial taxa with enlarged relative abundance in patients in Fig. 2d, *Veillonella*, *Campylobacter*, *R. mucilaginosa*, *Granulicatella*, *Kingella*, and *Filifactor* belongs to a group of periodontitis-correlated taxa, adding evidence to support the previous work denotative of a close relationship between periodontitis and SARS-CoV-2 infection^29^. Periodontal-associated cytokines may proliferate contacts of bacteria between the lungs and the mouth via driving the alteration of the respiratory epithelium and thereby promote respiratory infection in COVID-19 patients^14^. *H. parainfluenzae* and *Kingella*, two enriched taxa in the oral cavity of COVID-19 patients, are opportunistic pathogens well-known for respiratory tract infections, infective endocarditis, and meningitis^30,31^. The abrupt loss of *Neisseria* in patients, which is the highly abundant genus in the normal oral cavity, could raise serious damage to the oral microbiota^32,33^. In addition, all increased bacteria were previously classified as bacteremia-associated bacteria, implying a potential association between oral dysbiosis and secondary bacterial infection in COVID-19 patients^34^. Typically, mechanical ventilation supports may predispose the COVID-19 patients with severe symptoms of dyspnea to pulmonary bacterial co-infection, as the penetration of the device provides an entrance for those opportunistic infectious agents to access the lower respiratory tract from the oral cavity. Given the non-ignorable association between the oral microbiome with bacterial co-infections suggested by our and others’ data^35,36^, correct and frequent oral health care measurements should be recommended by physicians to protect COVID-19 patients from secondary infections and improve survival, especially for the ones with the severe disease condition.

Elevated levels of *Streptococcus*, *Rothia*, and *Actinomyces* were identified in COVID-19 patients’ feces, which is consistent with previous findings^37^. The host immunity symbionts beneficial bacterial species *B. obeum*, whose parent genus *Blautia* was listed as the top decreased taxon in COVID-19 patients’ feces in our data, was identified to be depleted in another study^17^. Notably, two normal components of the respiratory tract flora, *Granulicatella,* and *R. mucilaginosa*, were identified enriched in patients’ feces, which is likely a reflection of the migration of extra-intestinal microbes into the gut or the flourishment of potentially pathogenic bacteria. Typically, the fecal enrichment of specific oral taxa, which was suggested to be linked with the increased oral–fecal microbial transmission, has frequently been regarded as a hallmark of disease^20^. As SARS-CoV-2 viral RNA was detected in stools, the route of viral transmission from the respiratory tract to the intestinal tract can be hinted by the path of the oral flora translocation and colonization^38^. Moreover, *R. mucilaginosa* seemed further associated with the disease severity in the fecal samples in our study. As some severe patients with COVID-19 linked with cardiovascular disease comorbidities, this finding supports previous work wherein patients with cardiovascular disease comorbidities tended to have a higher prevalence of the *Rothia* ASV associated with SARS-CoV-2^39,40^. In addition, increased expression of ACE2 receptor was observed in *B. longum* treated mice; hence changes of *B. longum* might impact the individual’s susceptibility to SARS-CoV-2 through modulating ACE2 expression^41^. It is worth noticing that a decreased relative abundance of *B. caccae* and *B. coprophilus* was observed in the gut flora of COVID-19 patients. A recent study by Martino et al.^42^, has demonstrated that *Bacteroides* may regulate viral adhesion via modifying heparan sulfate (HS), and loss of HS-modifying *Bacteroides* strains could predispose individuals to SARS-CoV-2 infection. Concordance of these findings with ours provides evidence for a potential relationship between *B. caccae* and *B. coprophilus* and susceptibility to SARS-CoV-2 infection.

The Mantel and Procrustes analysis indicated the predicted function profiles aligned well with the bacterial taxonomy profile in both oral and fecal samples as expected. Compared to the gut microbiome, our analysis suggested a stronger impairment in the oral microbiome after SARS-CoV-2 infection, wherein a significant reduction of *Neisseria*, an essential oral microbial genus, was found, along with several fatal metabolic pathways involving the TCA cycle were suppressed. The result echoes a previous study that the gut microbial community possesses higher taxa-function robustness over oral community^43^, since the bacterial community in the gut generally has a higher gene and functional redundancy in comparison to communities inhabiting other body sites.

Microbiome dysbiosis persists during the COVID-19 disease course, even after the viral clearance. Moreover, previous studies have revealed that some recovered patients were re-detectable positive for SARS-CoV-2 RNA after discharge^44^. The cause of re-detectable positive remains unclear. One possibility is that a prolonged detrimental effect on the microbiomes exerted by SARS-CoV-2 infection persists even after discharge, which might render convalescent patients susceptible to the residual viremia or reinfection via a more long-lasting dysfunction of the immune system. The microbiome recovery at the taxonomy level seemed to be slowly healed in the oral samples as the PT(−) group shifts towards the healthy controls group based on the dissimilarity distance. In contrast, in the gut samples, the PF(−) group shifts even away from the healthy controls (Fig. 4b). The inconsistency may be due to the period span of sample collection or microbiome characteristics within the oral and gut samples. In a previous metagenomics study, Zuo and colleagues suggested four negatively associated *Bacteroides* species could be involved in the downregulation of ACE2 expression, and positively regulated *E. bacterium 2_2_44A* may promote augmenting SARS-CoV-2 infection in the gut^17^. However, a similar analysis method in our study did not identify the same agents to be associated with the viral load of SARS-CoV-2 (Fig. 4c). Thus, we urge validation of the results that should be taken before recognizing the results.

It is well accepted that unnecessary use of antibiotics should be avoided in the episode of acute respiratory infections as antibiotics have no role in treatment. In reality, 67.9% of patients in our study were treated with antibiotics to prevent potential secondary bacterial co-infections at the early phase of the pandemic. Besides, antibiotics prescribing was elevated in patients with severe conditions as compared to non-severe patients. In a meta-analysis study, Langford has reported that 74.6% of COVID-19 patients received antimicrobial treatment^28^. Antibiotics treatment can not only eliminate pathogens but commensal microorganisms indiscriminately, which may lead to microbiota dysbiosis and antimicrobial resistance. To circumvent and minimize the effect of antibiotics on the microbiome analysis, we employed a method to count antibiotics treatment as an adjusting variable in the model or to stratify the data for only patients without antibiotics treatment. In addition, unsupervised dimensionality reduction results demonstrated that COVID-19 patients with and without antibiotics treatment shared similar bacterial community structures with a declined diversity in both oral and gut microbiome, shifting away from healthy individuals. It seems that the impacts derived from infection are overwhelming over that of the antibiotic treatment that may apply to the microbiome. The taxa differential analysis also supported this finding, that the differential taxa between the controls and the antibiotics-free patients were largely overlapped with those identified between the controls and all patients (Supplementary Tables 3 and 5).

Collectively, we reported the alterations in both oral and gut microbiomes of SARS-CoV-2 infected patients during hospitalization and made comprehensive analyses to evaluate their potential consequences and implication. The associations between microbial species with disease severity and viral load in patients have suggested the potential of microbiome-based intervention in the prevention and treatment of COVID-19. We believe that the data provides new knowledge with innovative perspectives for tackling and managing the ongoing COVID-19 pandemic.

## Methods

### Study subject and sample collection

A total of 53 COVID-19 patients and 76 healthy individuals were included in this study. Patients with suspected SARS-CoV-2 infection were confirmed after two sequential positive respiratory tract sample real-time RT-PCR results. Patients were kept hospitalized and under strict observation until the virus was completely eliminated in both respirational and intestinal territories by real-time RT-PCR results. Depending on the sample availability, serial samples were collected from a patient throughout his/her hospitalization period. More specifically, throat swab and fecal samples were collected in both the positive viral RNA test period (P-VRTP, defined as the period of positive nucleic acid tests until the first day of continuous negative tests) and the negative viral RNA test period (N-VRTP, defined as the interval between the first day of negative nucleic acid test until the hospital discharge) for both sample types. Fecal samples were collected from patients who were ever detected with viral RNA in their feces. Only one sample, either throat swab or fecal specimen, was collected from healthy individuals during their physical examination. None of the COVID-19 patients was received antibiotics nor probiotics within 8 weeks before the infection, and none of the healthy individuals was either before this study recruitment. Patients were categorized into two groups based on disease severity: the non-severe group (mild/moderate) and the severe group (severe/critical) following the instruction of the New Coronavirus Pneumonia Prevention and Control Program (7th edition) published by the National Health Commission of China. The demographic information, underlying diseases, clinical indexes, and treatments were summarized from official patients’ medical records. This study was reviewed and approved by the Medical Ethical Committee of the Fifth Affiliated Hospital of Sun Yat-Sen University (approval # K162-1). Written informed consent was obtained from each enrolled subject.

### Sample library preparation and sequencing

Samples were inactivated at 56 °C for 30 min before DNA extraction. Extraction of nucleic acids was performed with the CFDA approved nucleic acid extraction kits (QIAamp Viral RNA Mini Kit, Catalog #: 52904, QIAGEN). The concentration and the purity were measured using the NanoDrop One (ThermoFisher Scientific, MA, USA). Sequencing libraries were generated using NEBNext® UltraTM II DNA Library Prep Kit for Illumina (New England Biolabs, MA, USA) following manufacturer’s recommendations, and index codes were added. The universal primers 338F (5′-ACTCCTACGGGAGGCAGCA-3′) and 806R (5′-GGACTACHVGGGTWTCTAAT-3′) were used for amplification of the V3–V4 region of the bacterial 16S rRNA gene. DNA libraries were generated from PCR amplicons targeting the hypervariable regions V3–V4 of the bacterial 16S rRNA gene. After quality assessment with the Qubit 2.0 Fluorometer (ThermoFisher Scientific, MA, USA), the library was then sequenced on an Illumina NovaSeq 6000 platform, and a minimum of 50,000 of 250 bp paired-end reads was generated for the samples.

### Bioinformatics analysis

Raw FASTQ files were demultiplexed using the QIIME 2 demux plugin based on their unique barcodes^45^. Demultiplexed sequences from each sample were stitched, quality filtered, trimmed, de-noised, and then the chimeric sequences were identified and removed using the QIIME 2 dada2 plugin to obtain the feature table^46^. The QIIME 2 feature-classifier plugin was then used to align feature sequences to a pre-trained GREENGENES 13_8 99% database to generate the taxonomy table^47^. The data was rarefied prior to alpha and beta diversity analysis using a depth of 13,111 reads. Any contaminating mitochondrial and chloroplast sequences were filtered using the QIIME 2 feature-table plugin. Diversity metrics were calculated and plotted using the core-diversity plugin and the emperor plugin within QIIME 2^48^. The beta diversity significance among groups was examined with PERMANOVA and PERMDISP tests using QIIME 2 plugins and Vegan package in R (version 4.0.2). The differences in the relative abundance of taxa between the patients and healthy control groups were identified in both sample types separately using the linear discriminant analysis effect size (LEfSe)^49^. Given the possible confounding impact, the output from LEfSe was further validated with Multivariate Association with Linear Models (MaAsLin2) by adjusting confounding factors (age, sex, antibiotic usage, PCR detection result, and patient ID), so only the bacteria taxa agreed by both methods were presented^50^. PICRUSt2 was used to predict the microbial metabolic pathways to assess the potential functional implication^51^. QIIME 2 Procrustes plugin was used to examine the fitness of the functional properties and the bacterial composition in the microbiome community with the Procrustes plot, and the correlation between functional properties (Bray Curtis distance) and the bacterial composition (unweighted UniFrac distance) in the microbiome community was examined by two-sided Mantel test.

### Detection of SARS-CoV-2 viral load

SARS-CoV-2 viral loads in the throat and fecal swabs were measured using a real-time RT-PCR assay. Viral RNA from throat swabs and fecal samples were extracted using QIAamp Viral RNA Mini Kit (QIAamp Viral RNA Mini Kit, Catalog #: 52904, QIAGEN). Up to 0.1 g of stool or throat swab was suspended in a 2 mL viral transport medium (in 1:10 dilution), followed by centrifugation at 3000 × *g* for 30 min. The aliquot of the filtrate was used as the starting material. The real-time RT-PCR was carried out with the Novel Coronavirus (2019-nCoV) real-time RT-PCR kit from LifeRiver Ltd. (Catalog #: RR-0479-02). Nucleocapsid gene (N), membrane gene (E), and RNA dependent RNA polymerase gene (RdRp) were the three targeted genes simultaneously amplified and tested. According to the manufacturer’s instructions, a combined result of the three SARS-CoV-2 viral gene targets was used to yield a positive result. Samples were considered negative if the cycle threshold values exceeded 43 cycles. Plasmids containing the full N gene and E gene were obtained to assess SARS-CoV-2 viral copy in the samples (PCDNA6B-SARS-CoV-2-N and PCDNA6B-SARS-CoV-2-E, gifts from Peihui Wang, Cheeloo College of Medicine, Shandong University). Serial 10-fold dilutions of known copies of these plasmids were prepared separately for generating the standard curve. The Ct values of real-time RT-PCR from patient samples were converted into viral RNA copies based on a standard curve.

### Statistical analysis

Categorical variables were presented as numbers and percentages (n/N, %) whereas continuous variables were reported as median and interquartile ranges (IQR). According to the distribution of data sets, the one-way analysis of variance (ANOVA) followed by Tukey’s multiple comparisons test or the Kruskal–Wallis test followed by Dunn’s multiple comparisons test were performed using Prism 8.3.0 (GraphPad Software). Given the nature of compositional data for the 16S microbial relative abundance information, we implemented CCLasso to define the correlation between the longitudinal viral load and the timepoint-matched relative abundances of different taxa in the throat swab and fecal samples separately^52^. The Spearman rank test was used to calculate the correlation between the viral load represented by the abundances of the N gene and the E gene, as their values did not follow the normal distribution according to the Kolmogorov–Smirnov test. All statistical tests were two-sided, and a *p*-value, of <0.05 was considered significant. Statistical analysis was performed using SPSS version 25.0 (SPSS Inc). Additional custom R scripts were used to making the plots.

### Reporting summary

Further information on research design is available in the Nature Research Reporting Summary linked to this article.

## Supplementary information

Supplementary Information

Reporting Summary

## Data Availability

Raw FASTQ files of 16S rRNA gene sequences were archived in the Sequence Read Archive (SRA) under Bioproject accession number PRJNA684070. All supporting data are publicly available at https://github.com/XiaominCheng/COVID19-16S.

## Code availability

The code used for analyses is publicly available at https://github.com/XiaominCheng/COVID19-16S.
