# Author Correction: Altered oral and gut microbiota and its association with SARS-CoV-2 viral load in COVID-19 patients during hospitalization

**DOI:** 10.1038/s41522-021-00262-z

**Published:** 2021-12-15

**Authors:** Yongjian Wu, Xiaomin Cheng, Guanmin Jiang, Huishu Tang, Siqi Ming, Lantian Tang, Jiahai Lu, Cheng Guo, Hong Shan, Xi Huang

**Affiliations:** 1 Center for Infection and Immunity, The Fifth Affiliated Hospital, Sun Yat-sen University, Zhuhai, Guangdong China; 2 School of Public Health, Sun Yat-sen University, Guangzhou, Guangdong China; 3 Guangdong Provincial Engineering Research Center of Molecular Imaging, Guangdong Provincial Key Laboratory of Biomedical Imaging, and Department of Interventional Medicine, The Fifth Affiliated Hospital, Sun Yat-sen University, Zhuhai, Guangdong China; 4 Southern Marine Science and Engineering Guangdong Laboratory, Zhuhai, Guangdong China; 5 National Clinical Research Center for Infectious Disease, Shenzhen Third People’ s Hospital; The Second Affiliated Hospital of Southern University of Science and Technology, Shenzhen, Guangdong China; 6 Department of Clinical Laboratory, The Fifth Affiliated Hospital of Sun Yat-sen University, Zhuhai, Guangdong China; 7 Center for Infection and Immunity, Mailman School of Public Health, Columbia University, New York, NY USA

**Keywords:** Clinical microbiology, Microbiome, Clinical microbiology, Microbiome

Correction to: *npj Biofilms and Microbiomes* 10.1038/s41522-021-00232-5, published online 22 July 2021

In this article the author name Guanmin Jiang was incorrectly written as Guangmin Jiang and the affiliation ‘Department of Clinical Laboratory, The Fifth Affiliated Hospital of Sun Yat-sen University, Zhuhai, Guangdong, China’ was missing.

The original article has been corrected.

