# Supplementary material for: Altered oral and gut microbiota and its association with SARS-CoV-2 viral load in COVID-19 patients during hospitalization
Source: NPJ Biofilms Microbiomes. 2021 Jul 22;7:61. doi: 10.1038/s41522-021-00232-5 (PMC8298611; doi:10.1038/s41522-021-00232-5)
Supplement: Supplementary file 1 — Supplementary Information [file 41522_2021_232_MOESM1_ESM.pdf]

Altered oral and gut microbiota and its association with SARS-CoV-2 viral load in COVID-19 patients during hospitalization

Yongjian Wu[1, 2, 3, 4, 5, +], Xiaomin Cheng[2, +], Guangmin Jiang[1, 3, +], Huishu Tang[1, 3], Siqi Ming[1, 3, 5], Lantian Tang[1, 3], Jiahai Lu[2, *], Cheng Guo[6,*], Hong Shan[3, 4, *], and Xi Huang[1, 3, 4, 5,*]

[1] Center for Infection and Immunity, the Fifth Affiliated Hospital, Sun Yat-sen University, Zhuhai, Guangdong Province, 519000, China

[2] School of public health, Sun Yat-sen University, Guangzhou, Guangdong Province, 510080, China

[3] Guangdong Provincial Engineering Research Center of Molecular Imaging, Guangdong Provincial Key Laboratory of Biomedical Imaging, and Department of Interventional Medicine, the Fifth Affiliated Hospital, Sun Yat-sen University, Zhuhai, Guangdong Province, 519000, China

[4] Southern Marine Science and Engineering Guangdong Laboratory, Zhuhai, Guangdong Province, 519000, China.

[5] National Clinical Research Center for Infectious Disease, Shenzhen Third People’ s Hospital; the Second Affiliated Hospital of Southern University of Science and Technology, Shenzhen, Guangdong Province, 518112, China.

[6] Center for Infection and Immunity, Mailman School of Public Health, Columbia University, New York, NY, 10032, USA

[+] These authors contributed equally to this work.

* Corresponding author:

Xi Huang, Email: huangxi6@mail.sysu.edu.cn

Hong Shan, Email: shanhong@mail.sysu.edu.cn

Cheng Guo, Email: cg2984@cumc.columbia.edu

Jiahai Lu, Email: lujiahai@mail.sysu.edu.cn

Supplementary Table 1. Samples and subjects characteristics by sample types

Group	PT	HT	PF	HF
**Samples characteristics**				
Sample collected	140	44	81	32
Viral RNA nucleic acid test, n/N (%)				
Viral RNA positive	52/140 (37.1)	/	50/81 (61.7)	/
Viral RNA negative	88/140 (62.9)	/	31/81 (38.3)	/
**Subjects characteristics**				
Individual enrolled	52	44	36	32
Gender				
Female, n/N (%)	22/52 (42.3)	13/44 (29.5)	13/36 (36.1)	11/32 (34.4)
Male, n/N (%)	30/52 (57.7)	31/44 (70.5)	23/36 (63.9)	21/32 (65.6)
Median age, y (IQR)	48.5 (32.0-64.0)	41.5 (36.3-51.0)	45.0 (32.0-63.5)	46.5 (42.3-53.0)
Antibiotic therapy, n/N (%)				
Yes	35/52 (67.3)	/	23/36 (63.9)	/
No	17/52 (32.7)	/	13/36 (36.1)	/
Antivirus therapy, n/N (%)		/		/
Yes	45/52 (86.5)	/	33/36 (91.7)	/
No	7/52 (13.5)	/	3/36 (8.3)	/
Disease severity, n/N (%)		/		/
Severe patients	22/52 (42.3)	/	10/36 (27.8)	/
Non-severe patients	30/52 (57.7)	/	26/36 (72.2)	/

Supplementary Table 2. Significantly distinct bacterial taxa identified between PT and HT groups

Taxanomy	Group	LEfSe analysis		MaAsLin2	
		LDA	p-value	Coefficient	p-value
Haemophilus_parainfluenzae	PT	4.058623	6.36E-06	0.125162812	0.001951781
g__Veillonella	PT	3.85207	3.05E-05	0.09671795	0.000675973
g__Campylobacter	PT	3.745349	4.85E-10	0.048989136	0.031114751
Rothia_mucilaginosa	PT	3.689566	1.62E-07	0.096630977	0.001737089
g__Granulicatella	PT	3.63394	6.66E-14	0.118068456	2.48E-09
Neisseria_subflava	PT	3.515048	5.66E-15	0.08987035	0.000444571
g__Kingella	PT	2.418025032	0.017809877	0.027200862	0.001758148
g__Filifactor	PT	2.073658868	0.003775443	0.019049393	0.019959509
g__Neisseria	HT	4.472161	8.38E-10	0.176739452	0.002368994
g__Corynebacterium	HT	4.09779	3.77E-09	0.145215834	0.001622564
g__Actinobacillus	HT	3.298003	8.70E-08	0.060967757	0.001859568
g__Moryella	HT	2.977811	0.000114	0.049638066	2.08E-05
g__Aggregatibacter	HT	2.952906432	0.000193928	0.046915302	0.002470034
Prevotella_intermedia	HT	2.88642374	0.000239621	0.057586129	0.00011425
g__Treponema	HT	2.84726	4.07E-05	0.068294957	0.000158995
g__Pseudomonas	HT	2.371535	6.24E-06	0.01469195	0.020469811
Treponema_amylovorum	HT	2.2748496	0.000737184	0.02417771	0.004387391

Supplementary Table 3. Significantly distinct bacterial taxa identified between PT(NS) and PT(S) groups as well as PT(abx-) and HT groups

Taxanomy	Group	LEfSe analysis		MaAsLin2	
		LDA	p-value	Coefficient	p-value
**PT(NS) vs. PT(S)**					
g__Treponema	PT(NS)	2.984098	5.17E-05	0.048845779	0.002277567
g__Aggregatibacter	PT(NS)	2.900273	1.43E-07	0.038650386	0.001778637
Aggregatibacter_segnis	PT(NS)	2.866217	5.00E-06	0.037485104	0.002347917
Prevotella_intermedia	PT(NS)	2.844975	3.00E-06	0.052287605	6.30E-06
Streptococcus_anginosus	PT(NS)	2.67914	0.002821	0.036319592	9.70E-05
g__Selenomonas	PT(NS)	2.67119	0.001722	0.027628854	0.004508061
g__Filifactor	PT(NS)	2.499093	6.39E-05	0.020426422	0.003365447
Treponema_amylovorum	PT(NS)	2.465991	0.001781	0.020520184	0.001860472
g__Peptococcus	PT(NS)	2.276831	0.002267	0.017592997	0.003143184
**PT(abx-) vs. HT**					
Neisseria_subflava	PT(abx-)	3.772602791	7.55E-16	0.134291103	0.001160573
g__Granulicatella	PT(abx-)	3.728665341	5.55E-14	0.089398883	0.000787813
g__Veillonella	PT(abx-)	3.708256093	5.53E-05	0.149621381	9.72E-05
g__Campylobacter	PT(abx-)	3.688631824	6.16E-06	0.140870823	0.000120171
Rothia_mucilaginosa	PT(abx-)	3.461218724	6.00E-08	0.15283721	6.75E-07
Veillonella_dispar	PT(abx-)	3.383724644	0.03595859	0.150075686	0.001992298
g__Peptoniphilus	PT(abx-)	2.428251	0.002413	0.035286204	0.009795435
g__Moryella	HT	3.086687563	5.56E-05	0.055519744	0.018107181
g__Treponema	HT	2.922628735	0.001077494	0.076820179	0.004423529
Prevotella_intermedia	HT	2.8154732	0.004906798	0.065129511	0.011724276

Supplementary Table 4. Significantly distinct bacterial taxa identified between PF and HF groups

Taxanomy	Group	LEfSe analysis		MaAsLin2	
		LDA	p-value	Coefficient	p-value
g__Streptococcus	PF	4.07039439	9.81E-06	0.294723786	0.000230565
g__Weissella	PF	3.517819013	8.26E-05	0.101335921	0.014669427
g__Enterococcus	PF	3.199232248	2.80E-05	0.089347099	0.000623727
Clostridium_citroniae	PF	3.088746	5.85E-05	0.052788354	0.007081948
Bifidobacterium_longum	PF	2.976288515	7.88E-07	0.044654773	0.017956597
Rothia_mucilaginosa	PF	2.947328878	0.000177757	0.079359876	1.08E-05
g__Rothia	PF	2.939548147	0.002789322	0.076703639	1.77E-05
g__Lactobacillus	PF	2.831048869	0.017048742	0.065256175	0.004378413
g__Actinomyces	PF	2.536534912	0.000329375	0.038366765	0.002006929
g__Granulicatella	PF	2.397197	3.41E-06	0.024857664	0.002892692
g__Blautia	HF	3.836604	1.39E-12	0.123614968	3.67E-05
g__Coprococcus	HF	3.619681	1.16E-12	0.100262772	1.59E-05
Bacteroides_caccae	HF	3.118124487	4.02E-09	0.07527124	0.004790875
Bacteroides_coprophilus	HF	2.859626	7.36E-16	0.034969496	0.017497726
Blautia_obeum	HF	2.777926	2.03E-11	0.046428815	0.000573894
Clostridium_colinum	HF	2.458573	6.52E-15	0.024786391	0.0016486
g__Collinsella	HF	2.209255	2.68E-05	0.03146611	0.018499252

Supplementary Table 5. Significantly distinct bacterial taxa identified between PF(abx-) and HF groups

Taxanomy	Group	LEfSe analysis		MaAsLin2	
		LDA	p-value	Coefficient	p-value
g__Streptococcus	PF(abx-)	3.693343	0.000352	0.090926687	0.003154147
Clostridium_citroniae	PF(abx-)	3.268273	1.10E-05	0.05066731	0.004119379
g__Anaerococcus	PF(abx-)	3.070141	0.001968	0.055366168	0.000344614
Streptococcus_anginosus	PF(abx-)	3.032709	0.002302	0.04131649	0.002373282
g__Peptoniphilus	PF(abx-)	3.016881	0.000433	0.053420717	0.000245755
g__Campylobacter	PF(abx-)	2.533064	0.048023	0.024358408	0.004861695
g__Atopobium	PF(abx-)	2.350014	0.000433	0.008465937	0.009964554
g__Blautia	HF	3.864694	2.73E-09	0.139410699	6.99E-08
g__Coprococcus	HF	3.581713	2.71E-07	0.084764896	1.81E-05
Bacteroides_eggerthii	HF	3.064006	1.04E-08	0.056029014	0.001243332
Bacteroides_coprophilus	HF	2.853866	3.17E-07	0.037224891	0.00847245
Blautia_obeum	HF	2.812043	7.68E-06	0.044527621	0.000173239
Blautia_producta	HF	2.76882	0.00015	0.036507669	0.003013506
g__Anaerostipes	HF	2.686756	0.000189	0.032725558	0.002400145
Clostridium_colinum	HF	2.514328	1.20E-07	0.022969211	0.001295239
g__Neisseria	HF	2.501995	2.71E-06	0.02405077	0.003672718

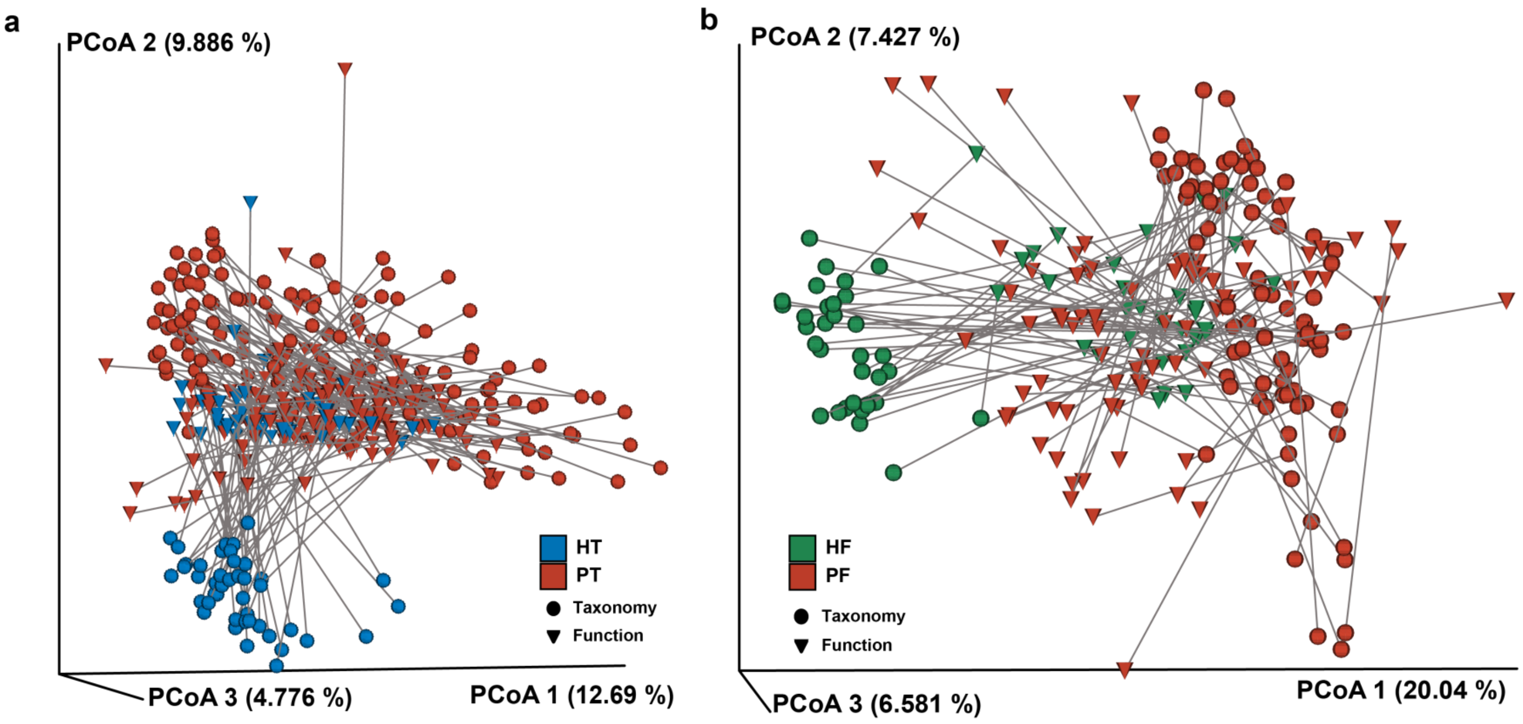

Supplementary Fig. 1 Procrustes analyses demonstrated a significant correlation between the potential function profile and microbial composition. a Oral microbiota. **b** Gut microbiota. PT group and HT group are colored in red and blue, respectively; PF group and HF group are colored in red and green, respectively. The squares indicate 16S rRNA gene OTU data of microbial composition, and the triangles indicate the potential function profile.

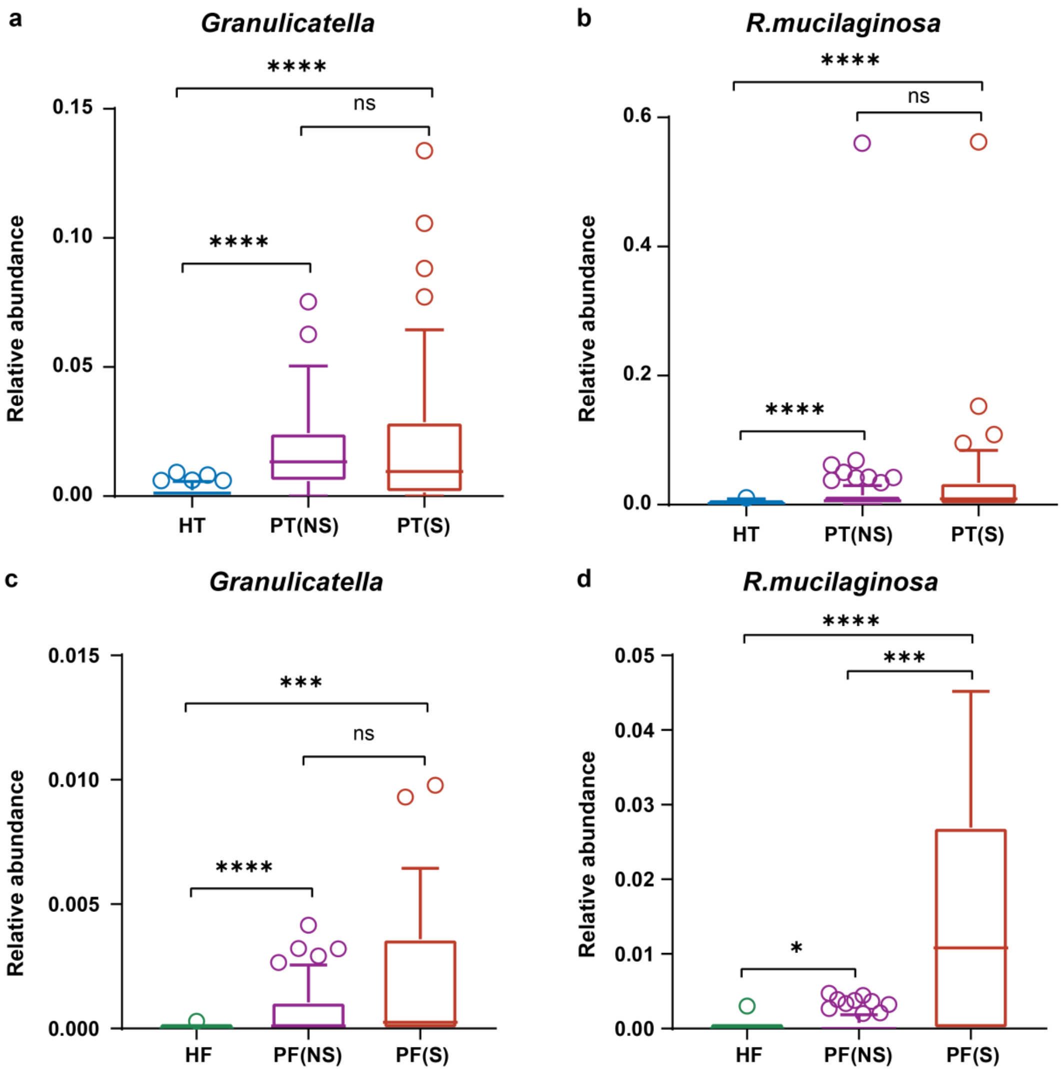

Supplementary Fig. 2 Relative abundance of the oral and gut microbiota among the healthy controls, patients with the non-severe condition, and patients with the severe condition. a Relative abundance of *Granulicatella* among HT, PT(NS) and PT(S). **b** Relative abundance of *R. mucilaginosa* among HT, PT(NS) and PT(S). **c** Relative abundance of *Granulicatella* among HF, PF(NS) and PF(S). **d** Relative abundance of *R. mucilaginosa* among HF, PF(NS) and PF(S). ns: not significant, *$p < 0.05$, *** $p < 0.001$, ****$p < 0.0001$. p values were obtained using the Kruskal-Wallis test followed by Dunn's multiple comparisons test. The line in the middle of the box, bound of the box and whiskers represent the median, 25th-75th percentiles and min-to-max values, respectively.
