# Supplementary material for: Altered oral and gut microbiota and its association with SARS-CoV-2 viral load in COVID-19 patients during hospitalization
Source: NPJ Biofilms Microbiomes. 2021 Jul 22;7:61. doi: 10.1038/s41522-021-00232-5 (PMC8298611; doi:10.1038/s41522-021-00232-5)
Supplement: Supplementary file 2 — Reporting Summary [file 41522_2021_232_MOESM2_ESM.pdf]

nature research

Corresponding author(s): Prof. Xi Huang
Prof. Hong Shan
Dr. Cheng Guo
Prof. Jiahai Lu

Last updated by author(s): Jun 10, 2021

Reporting Summary

Nature Research wishes to improve the reproducibility of the work that we publish. This form provides structure for consistency and transparency in reporting. For further information on Nature Research policies, see our Editorial Policies and the Editorial Policy Checklist.

Statistics

For all statistical analyses, confirm that the following items are present in the figure legend, table legend, main text, or Methods section.

n/a	Confirmed	
☐	☒	The exact sample size (n) for each experimental group/condition, given as a discrete number and unit of measurement
☐	☒	A statement on whether measurements were taken from distinct samples or whether the same sample was measured repeatedly
☐	☒	The statistical test(s) used AND whether they are one- or two-sided<br>*Only common tests should be described solely by name; describe more complex techniques in the Methods section.*
☐	☒	A description of all covariates tested
☐	☒	A description of any assumptions or corrections, such as tests of normality and adjustment for multiple comparisons
☐	☒	A full description of the statistical parameters including central tendency (e.g. means) or other basic estimates (e.g. regression coefficient) AND variation (e.g. standard deviation) or associated estimates of uncertainty (e.g. confidence intervals)
☐	☒	For null hypothesis testing, the test statistic (e.g. F, t, r) with confidence intervals, effect sizes, degrees of freedom and P value noted<br>*Give P values as exact values whenever suitable.*
☒	☐	For Bayesian analysis, information on the choice of priors and Markov chain Monte Carlo settings
☒	☐	For hierarchical and complex designs, identification of the appropriate level for tests and full reporting of outcomes
☐	☒	Estimates of effect sizes (e.g. Cohen's d, Pearson's r), indicating how they were calculated

Our web collection on statistics for biologists contains articles on many of the points above.

Software and code

Policy information about availability of computer code

Data collection	The 16S rRNA sequencing data were generated via the Illumina NovaSeq 6000 platform. Viral RNA from throat swabs and fecal samples were extracted using QIAamp Viral RNA Mini Kit (QIAamp Viral RNA Mini Kit, Catalog #: 52904, QIAGEN). The real-time RT-PCR was carried out with the Novel Coronavirus (2019-nCoV) real-time RT-PCR kit from LifeRiver Ltd. (Catalog #: RR-0479-02).
Data analysis	The followings were used for data analysis:<br>QIIME2 with available tutorial pipeline<br>R version 4.0.2<br>ggplot2 package, openxlsx package, ggrepel package, tidyr package, grid package, vegan package, EcolUtils package in the R<br>MaAsLin2<br>LEfSe with available tutorial pipeline<br>Prism 8.3.0 (GraphPad Software)<br>SPSS version 25.0 (SPSS Inc)

For manuscripts utilizing custom algorithms or software that are central to the research but not yet described in published literature, software must be made available to editors and reviewers. We strongly encourage code deposition in a community repository (e.g. GitHub). See the Nature Research guidelines for submitting code & software for further information.

Data

Policy information about availability of data

All manuscripts must include a data availability statement. This statement should provide the following information, where applicable:

- Accession codes, unique identifiers, or web links for publicly available datasets
- A list of figures that have associated raw data
- A description of any restrictions on data availability

All supporting data are within the paper and its additional files. Raw FASTQ files of 16S rRNA gene sequences were archived in the Sequence Read Archive (SRA) under Bioproject accession number PRJNA684070 (https://www.ncbi.nlm.nih.gov/bioproject/PRJNA684070). The data and code used for analyses are publicly available at https://github.com/XiaominCheng/COVID19-16S.

Field-specific reporting

Please select the one below that is the best fit for your research. If you are not sure, read the appropriate sections before making your selection.

☒ Life sciences ☐ Behavioural & social sciences ☐ Ecological, evolutionary & environmental sciences

For a reference copy of the document with all sections, see nature.com/documents/nr-reporting-summary-flat.pdf

Life sciences study design

All studies must disclose on these points even when the disclosure is negative.

Sample size	Sample size for the COVID-19 patients was largely determined by the sample availability. To ensure the statistics power, the maximum number of study subject was considered as long as the individual meets the inclusion criteria of the study. At least 32 individuals were included in each sample type group category (Sup Table 1). A total of 140 throat swab samples and 81 fecal samples from these COVID-19 patients during hospitalization, as well as 44 throat swab samples and 32 fecal samples from sex and age matched healthy individuals were collected for the study.
Data exclusions	No data were excluded from the analyses.
Replication	Depending on the sample availability, serial samples were collected from a patient throughout his/her hospitalization period.
Randomization	The allocation of participants is not random that we include all available COVID-19 patients who meet the inclusion criteria. When conducting relevant analyses, confounding covariates (age, sex, antibiotic use, PCR detection result, and patient ID) were adjusted, which is indicated in the manuscript as well.
Blinding	Blinding was not performed because the study is retrospective and the experimental design did not include specific intervention.

Reporting for specific materials, systems and methods

We require information from authors about some types of materials, experimental systems and methods used in many studies. Here, indicate whether each material, system or method listed is relevant to your study. If you are not sure if a list item applies to your research, read the appropriate section before selecting a response.

Materials & experimental systems

n/a	Involved in the study
☒	☐ Antibodies
☒	☐ Eukaryotic cell lines
☒	☐ Palaeontology and archaeology
☒	☐ Animals and other organisms
☐	☒ Human research participants
☒	☐ Clinical data
☒	☐ Dual use research of concern

Methods

n/a	Involved in the study
☒	☐ ChIP-seq
☒	☐ Flow cytometry
☒	☐ MRI-based neuroimaging

Human research participants

Policy information about studies involving human research participants

Population characteristics	The sample distribution, demographics, and relevant clinical information about the subjects recruited in the study was summarized in Supplementary Table 1.
Recruitment	Patients with suspected SARS-CoV-2 infection were confirmed after two sequential positive respiratory tract sample real-time

Recruitment	RT-PCR results. Patients were kept hospitalized and under strict observation until the virus was completely eliminated in both respirational and intestinal territories by real-time RT-PCR results. Depending on the sample availability, serial samples were collected from a patient throughout his/her hospitalization period. More specifically, throat swab and fecal samples were collected in both the positive viral RNA test period (P-VRTP, defined as the period of positive nucleic acid tests until the first day of continuous negative tests) and the negative viral RNA test period (N-VRTP, defined as the interval between the first day of negative nucleic acid test until the hospital discharge) for both sample types. Fecal samples were collected from patients who were ever detected with viral RNA in their feces. Only one sample, either throat swab or fecal specimen, was collected from healthy individuals during their physical examination. None of the COVID-19 patients was received antibiotics nor probiotics within 8 weeks before the infection, and none of the healthy individuals was either before this study recruitment. Patients were categorized into two groups based on disease severity: the non-severe group (mild/moderate) and the severe group (severe/critical) following the instruction of the New Coronavirus Pneumonia Prevention and Control Program (7th edition) published by the National Health Commission of China. The demographic information, underlying diseases, clinical indexes, and treatments were summarized from official patients' medical records.
Ethics oversight	This study was reviewed and approved by the Medical Ethical Committee of the Fifth Affiliated Hospital of Sun Yat-Sen University (approval # K162-1). Written informed consent was obtained from each enrolled subject.

Note that full information on the approval of the study protocol must also be provided in the manuscript.
